# Perfecting the Plaster Cast*Extracted from *Dental Summary*, May, 1921.

**Published:** 1921-12

**Authors:** I. L. Furnas


### PERFECTING THE PLASTER CAST*

BY I. L. FURNAS

This is an outline of the technic that I believe to be highly commendable, using for the cast a material known as Spence's Plaster Compound.

Spence's Plaster Compound when properly mixed has a crushing resistance of 1,000 pounds per square inch, 24 hours after mixing, and 1,800 pounds per square inch, 12 hours after mixing. Notice, please, that there is a difference of about 800 pounds before and after the mix has gone through the sweating stage. This should tell us that our Spence Plaster Compound casts should be used, if possible, before they have gone through this sweating stage.

The deterioration of Spence's Plaster Compound casts during the process of vulcanization begins at approximately 300 degrees Fahrenheit, or at about the point where the flow of most rubbers in the flask ceases. This is very desirable because it is very important that our matrix should retain its shape until our denture is fully formed.

This disintegration advances with the heat up to 340 degrees Fahrenheit, at which time it has reached a disintegration point of about 60 per cent.

I have been able to register about two one-thousandths of an inch contraction in Spence's Plaster Compound, but never any expansion; however, in my estimation this change is so small that it is of very minor importance.

---

* Extracted from *Dental Summary*, May, 1921.

Inasmuch, then, as compression is one of the chief requisites of a cast material, and as Spence's Plaster Compound when properly mixed stands very high in compression, and inasmuch as it retains this characteristic after the flask is placed in the vulcanizer and the heat applied to a point of temperature past the rubber flow, then I believe you must agree that it is a good cast material.

There are few men who realize the enormous pressure applied on the face of a cast in closing a flask. Dr. Prothero, of Chicago, earned the credit for working this out years ago. He found by using fifty pounds pressure as an average on the end of a four-inch ordinary flask wrench, with a one-to-twenty-pitch ordinary flask bolt, the face of the cast was subjected to the most unbelievably terrific strain of over *four tons per square inch;* to be exact it was 8,333 pounds plus.

Does this one thing alone explain to any of you men why some of your dentures do not meet all your expectations as regards adaptation and antagonization?

Spring pressure for flask closing is correcting this trouble to a marked degree, but even yet we must exercise more care in our flask closing.

The technic for constructing *casts* is simple and to most of the profession old.

The back of the impression should be imbedded in a heavy mix of plaster of paris which serves as a foundation and reinforcement. This should be about one-eighth-inch thick at its thinnest place, and averaging about one-quarter-inch in thickness. It should be trimmed so that its edges will extend laterally and at right angles to the margins of the impression for about one-eighth of an inch.

Next is added a layer of modeling clay which is carried to the turn established by the muscle-trimming and at right angles.

This modeling clay is much better for this work than plaster of paris because it has no expansion or contraction and is much more easily adapted to the borders of the turn on the impression, and eliminates the danger of breaking frail margins.

Upon and around this reinforced impression a strip of paraffin wax is sealed at the base of the reinforcement so as to be water-tight boxed impression. This wax border is next trimmed for height. This is easily determined by placing a straight edge from border to border and trimming so that the thinnest part of the cast will be at least one-quarter-inch thick. This wax border may be still further reinforced with plaster of paris if the wax is very thin. In place of using the paraffin wax for this part of the boxing some men use waxed linen strips, tea-lead strips, cardboard, and other similar things.

The boxed impression is now coated with a solution of shellac in alcohol, and as soon as it is dry, paint it with a fifteen per cent solution of sandarac in alcohol. When this coating has thoroughly hardened, fill with soap suds, using a heavy laundry soap in preference to some of the lighter toilet soaps. This soap is then immediately flushed out, but a sufficient amount will remain to act as a separating medium between the cast and the separating mediums proper, and preventing them from sticking to the face of the cast.

The boxing is now filled with water and permitted to stand for from five to ten minutes. This water is now poured into the plaster bowl and will be found to be about the correct amount to use for the proper mix of the Spence Plaster Compound for this particular case.

The Spence Plaster Compound which is now added to this water will make a heavy dry mix. This should be kneaded in the soft rubber plaster bowl, using the hands in preference to a plaster spatula. When the mix attains a heavy, putty-like consistency it is taken from the plaster bowl and kneaded in the hands, using a rotating or sliding movement to insure the breaking up of any lumps of the material, insuring a smooth even mix. When it is thoroughly mixed, a small piece about the size of a hickory nut is pressed in the hands to expell all the air and then placed in the center of the impression. The case is then jarred or beaten up and down on the laboratory bench, table, or something similar, until the Spence Plaster Compound spreads and fills all the fine lines and details of the impression. More Spence Plaster Compound is added until the boxed impression is filled. There are many special lathe devices used in jarring this Spence Plaster Compound mix into place; but the simple method of beating it down as I have described has been much more satisfactory in my hands.

The cast may be separated in from three to five hours; however, whenever possible it is advisable to let it set longer or over night if convenient.—*Dental Summary,* May, 1921.

